# Achieving child-centred care for children and young people with life-limiting and life-threatening conditions—a qualitative interview study

**DOI:** 10.1007/s00431-022-04566-w

**Published:** 2022-08-12

**Authors:** Lucy Coombes, Debbie Braybrook, Anna Roach, Hannah Scott, Daney Harðardóttir, Katherine Bristowe, Clare Ellis-Smith, Myra Bluebond-Langner, Lorna K. Fraser, Julia Downing, Bobbie Farsides, Fliss E. M. Murtagh, Richard Harding, on behalf of C-POS

**Affiliations:** 1 Florence Nightingale Faculty of Nursing Midwifery and Palliative Care, Cicely Saunders Institute of Palliative Care, Policy and Rehabilitation, King’s College London, London, UK; 2 Royal Marsden NHS Foundation Trust, London, UK; 3 Louis Dundas Centre for Children’s Palliative Care, University College London, London, UK; 4 Martin House Research Centre, Department of Health Sciences, University of York, York, UK; 5 International Children’s Palliative Care Network, Kampala, Uganda; 6 Rutgers University, New Brunswick, NJ USA; 7 Brighton and Sussex Medical School, University of Sussex, Brighton, UK; 8 Wolfson Palliative Care Research Centre, Hull York Medical School, University of Hull, Hull, UK

**Keywords:** Paediatrics, Palliative care, Normality, End of life care, Children, Symptom assessment

## Abstract

This study aims to identify the symptoms, concerns, and care priorities of children with life-limiting conditions and their families. A semi-structured qualitative interview study was conducted, seeking perspectives from multiple stakeholders on symptoms, other concerns, and care priorities of children and young people with life limiting and life-threatening conditions and their families. Participants were recruited from six hospitals and three children’s hospices in the UK. Verbatim transcripts were analysed using framework analysis. A total of 106 participants were recruited: 26 children (5–17 years), 40 parents (of children 0–17 years), 13 siblings (5–17 years), 15 health and social care professionals, 12 commissioners. Participants described many inter-related symptoms, concerns, and care priorities impacting on all aspects of life. Burdensome symptoms included pain and seizures. Participants spoke of the emotional and social impacts of living with life-limiting conditions, such as being able to see friends, and accessing education and psychological support. Spiritual/existential concerns included the meaning of illness and planning for an uncertain future. Data revealed an overarching theme of pursuing ‘normality’, described as children’s desire to undertake usual childhood activities. Parents need support with practical aspects of care to help realise this desire for normality.

*Conclusion*: Children with life-limiting conditions and their families experience a wide range of inter-related symptoms, concerns, and care priorities. A holistic, child-centred approach to care is needed, allowing focus on pursuit of normal childhood activities. Improvements in accessibility, co-ordination, and availability of health services are required to achieve this.

**Table:** 

**What is Known:** *• Existing evidence regarding symptoms, concerns, and care priorities for children with life-limiting conditions is largely limited to proxy-reported data and those with a cancer diagnosis.* *• Child-centred care provision must be directed by children’s perspectives on their priorities for care. *	
**What is New:** *• Social and educational activities are more important to children with life-limiting conditions than their medical concerns.* *• A holistic approach to care is required that extends beyond addressing medical needs, in order to support children with life-limiting conditions to focus on pursuit of normal childhood activities.*

**Supplementary Information:**

The online version contains supplementary material available at 10.1007/s00431-022-04566-w.

## Introduction

Worldwide there are approximately 21 million children and young people aged 0–19 years (hereafter ‘children’) with life-limiting and life-threatening conditions (hereafter ‘life-limiting’) [1]. Life-limiting conditions are those for which there is no hope of cure and from which children will die [2]. Life-threatening conditions are those for which curative treatment may be feasible but may fail [2].

Due to medical advances, increasing numbers of children are living with life-limiting conditions [3, 4]. However, provision of children’s palliative care varies geographically, and increased prevalence has not been met with an equivalent increase in healthcare resource [3, 5].

Palliative care for adults is effective and cost-effective, reducing unplanned admissions and futile treatments [6–8], while improving quality of life, care quality, and survival [9–11]. There are almost 400 conditions that affect children for which palliative care could be beneficial [3, 12]. However, evidence for effectiveness of children’s palliative care is limited in part due to a lack of a valid and reliable outcome measure [13, 14]. Development of such a measure has repeatedly been identified as a research priority [15–17]. A measure is in development in sub-Saharan Africa and Belgium, but primary data to inform measurement has not been generated outside Africa [18–20].

Outcome measure development for children with life-limiting conditions is complex due to differences in age and developmental stage, the range of conditions [12], and the role of family in care provision. To establish face and content validity, it is imperative to understand which symptoms and concerns matter the most. However, most studies focus on children with cancer [21], or rely on proxy reports of parent/carers (hereafter ‘parents’) or health and social care (healthcare) professionals [21]. This exclusion of children from participating in primary research directly contradicts the growing focus on children having agency, with a right to be involved in their own healthcare decisions [22, 23] as active partners in their healthcare, not passive recipients [22, 24]. This study aimed to identify the symptoms, concerns, and care priorities of children with life-limiting conditions and their families.

## Methods

### Study design

Semi-structured, qualitative interview study reported in accordance with the consolidated criteria for reporting qualitative studies (COREQ) [25].

### Setting

Children, parents, and healthcare professionals were recruited from six hospitals and three children’s hospices within three UK countries.

Commissioners were recruited via recommendations from healthcare professionals and the UK’s national children’s palliative care advocacy charity.

### Sampling and recruitment

#### Inclusion criteria

Children (5–17 years) with any life-limiting condition; parents/carers with a child < 18 years old with a life-limiting condition; siblings (5–17 years) of children with a life-limiting condition; healthcare professionals with > 6 months experience of caring for children with life-limiting conditions; commissioners of UK paediatric palliative care services.

#### Exclusion criteria

Children: unable to communicate via an in-depth interview, using ‘draw and talk’ or play methods or via their parents; speaks a language not supported by NHS translation services; currently enrolled in another study; unable to give consent/assent.

Parents/carers and siblings: unable to give consent/assent, speaks a language not supported by NHS translation services.

Purposive sampling was used to ensure maximum variation in key demographics such as age and condition. Given the heterogeneity of the sample, the concept of pragmatic saturation was used to determine the required sample size in order for the dataset to have the required diversity and depth to meet the aims and objectives of the study [26].

### Data collection

Semi-structured interviews were conducted using a topic guide informed by a systematic review of symptoms and concerns in children with life-limiting conditions [21] and the World Health Organisation (WHO) definition of paediatric palliative care [27]. The topic guide was reviewed by the study steering group (healthcare professionals, parents, and researchers). Interviews were conducted by LC (experienced children’s palliative care nurse, new to qualitative research), AR (new to qualitative research), and DB (experienced qualitative researcher). All interviewers received training and supervision on conducting interviews with children, including communication, legal, and ethical issues.

Interviews commenced with demographic questions and children were asked about their hobbies and interests to build rapport. Play and drawing were used to aid interviews where required. The topic guide contained an open question asking participants to describe their/their child’s condition and how it affects their/their child’s life. Interviews with professionals asked about the main symptoms, concerns, and care priorities of children with life-limiting conditions. Probes ensured that all domains from the WHO definition of palliative care were discussed, while allowing participants to discuss what mattered most. Interviews were audio-recorded, transcribed verbatim, and pseudonymised.

### Data analysis

Transcripts were analysed by LC, DB, AR, DH, and HS using deductive (from the WHO domains of palliative care [27]) and inductive coding [28, 29]. Analysis followed the five steps of framework analysis: familiarisation, constructing a thematic framework, indexing and sorting, charting and mapping/interpretation [28–30] using NVivo software (Version 12). Using framework analysis allowed the authors to compare and contrast the findings from each theme overall and by participant group. Regular meetings were held to discuss emerging themes and resolve any differences (20% of transcripts were independently coded by two researchers). RH, KB, and CES were consulted if discrepancies could not be resolved. Analysis was reviewed by the study steering group throughout the study.

### Ethical approval

Ethical approval was granted by the Bloomsbury research ethics committee (HRA:19/LO/0033). Participants over 16 years old provided written informed consent. Those with parental responsibility provided written informed consent for participants < 16 years. Those < 16 years provided written assent.

## Results

### Participant characteristics

A total of 103 interviews were conducted (April 2019–September 2020) with 106 participants: 26 children, 40 parents, 13 siblings, 15 health and social care professionals and 12 commissioners (see Table 1). Two sets of parents and one set of siblings were interviewed together. ICD-10-chapter headings are reporting for pseudonymity as some children reported rare conditions. Most interviews were carried out face-to-face in a location of the participant’s choosing. Due to the COVID-19 pandemic, 13 interviews were conducted remotely (telephone or video call) [31].

**Table 1:** Demographic details of participants

	*n* or mean (range)
**Children (*n* = 26)**	
Age (yrs)	12 (5–17)
Gender	
* Female:male*	17:9
Diagnosis	
*Cancer* * Congenital* * Gastrointestinal* * Metabolic* * Neurological* * Respiratory*	6 3 10 1 5 1
Interview duration (mins)	37 (12–81)
**Parent/carers (*n* = 40)**	
Age (yrs)	40 (21–65)
Gender	
* Female:male*	30:10
Relationship to child	
* Mother* * Father*	30 10
Diagnosis of child	
* Cancer* * Congenital* * Gastrointestinal* * Genitourinary* * Infectious disease* * Metabolic* * Neurological* * Perinatal*	6 7 4 1 2 9 10 1
Age of child with life-limiting condition (years)	12 (0–17)
Interview duration (mins)	63 (33–161)
**Siblings (*n* = 13)**	
Age (yrs)	9 (5–15)
Gender	
* Female:male*	7:6
Diagnosis of child	
* Congenital* * Gastrointestinal* * Metabolic* * Neurological*	3 2 1 7
Age of child with life-limiting condition (yrs)	10 (3–16)
Interview duration (mins)	26 (8–37)
**Health and social care professionals (*n* = 15)**	
Gender	
* Female:male*	14:1
Profession	
Doctor* Nurse** * Social worker* * Chaplain* * Psychologist* * Play specialist* * Physiotherapist*	3 7 1 1 1 1 1
Interview duration (mins)	55 (38–82)
**Commissioners (*n* = 12)**	
Gender	
* Female:male*	11:1
Geographical location	
* Southeast England* * Greater London* * East England* * Northwest England* * Yorkshire and Humber*	4 1 2 1 4
Interview duration (mins)	53 (33–86)

*1 paediatric palliative medicine consultant, 1 haematology consultant, 1 general paediatrician

**4 palliative care nurse specialists, 1 children’s community nurse, 1 hospice nurse, 1 ward sister

### Priority healthcare outcomes

The priority healthcare outcomes of children with life-limiting conditions and their families fitted into five themes—physical, spiritual and existential, emotional and psychological, social and practical, and pursuing normality. Table 2 shows these themes and the subthemes that comprise them. Illustrative quotes are presented in Tables 3, 4, and 5 and supplementary Table 1 (S1). Themes and subthemes were often closely inter-related.

**Table 2:** Inter-related domains and themes—symptoms, concerns, and care priorities (*n* = 106)

**Themes**	**Pursuing normality**	**Physical**	**Spiritual/existential**	**Emotional/psychological**	**Social**	**Practical**
**Subthemes**	Not knowing any different	Pain	Life unlived	Awareness of difference	Loneliness and isolation	Minimising hospital stays—preventing unplanned admissions, timely discharge
	Regaining normality	Other symptoms, e.g., seizures, infection, breathing difficulties, nausea, and vomiting	Religious beliefs and needs	Need to meet others the same	Access to social support	Service provision and availability, e.g., 24/7 care at home, access to respite, care continuity and co-ordination, and facilities
	Adjusting to a new normal	Management of symptoms	Hopes for and uncertainty about the future	Control and independence,	Communication and decision making (including building trust and respect, managing discord, managing goals, and expectations)	Burden and logistics of care
		Medical interventions, e.g., minor procedures, surgery, feeding tube insertion, and blood tests	Living a full life	Protecting family members	Balancing needs of family	Information needs
		Eating and drinking	Determination to overcome condition	Emotions, e.g., worry and anger sadness	Employment, housing, and financial concerns	Changing needs
		Sleep, fatigue, and tiredness	Meaning of life	Memory making and wishes	Access to technology and social media	Advance care planning
		Changes in physical appearance		Loss of self-confidence	Enjoying usual childhood activities, e.g., hobbies, play, school, and friendships	Transitions (care settings, change of school, child and adult services)
				Impact on family life	Restrictions on day-to-day life	Access to equipment
				Psychological and emotional support		
				Memory making and wishes		
				Privacy and dignity

**Table 3:** Participant quotes—physical symptoms and concerns, and spiritual and existential concerns

**Quote number**	**Quote**	**Participant details**
**Physical symptoms and concerns**		
Q1	‘…and that’s what we live for, we just carry on for her smiles. Because she doesn’t have a great value of life, this is (child’s) life mainly but she is happy…erm and she’s not in discomfort, so I can’t really ask for anything more than that.’	Mother of an 8-year-old with a neurological condition
Q2	‘So, it’s about being realistic but reassuring them that we have different medications we can use for different situations and that we will continuously try and control symptoms. Obviously not promising that we can get everything under control, but we will try our hardest’	Nurse
Q3	‘Now it’s about trying to control seizures the best we can, we know we can’t totally control them’	Mother of a 14-year-old with a metabolic condition
Q4	‘P: Well, she has seizures and they’re triggered easily, pretty easily…umm I: Do you know what sort of things trigger them? P: Umm…her being excited, like going to do like a sport that will trigger it, like swimming that could…’	Sibling of a child with a neurological condition
Q5	They say if you don’t eat then you need a nose tube. I don’t like them	11-year-old with cancer
Q6	‘…erm or when we have anything from our treat box, it…I kind of feel sorry for him because he can’t…he’s watching us eat it and he can’t eat any of ‘em’	Sibling of a child with a gastrointestinal condition
Q7	‘its very difficult when people say ‘well can’t you just put him in his wheelchair and take him for a walk round the block?’ and I’m like ‘I haven’t slept for fourteen hours’. I don’t wanna get him in his chair and take him for a walk around the block because I…I’m exhausted and it’s not because I’m lazy, its because I’m physically exhausted’	Mother of 14-year-old with metabolic condition
Q8	‘I get worn out a lot quicker, so I can’t like run around for long or stand for long…. or like go on long walks’	14-year-old, congenital condition
Q9	‘…sometimes you see like, when you…when like you’re at the park or something, like you see people staring and you just think…oh honestly, I couldn’t really care any less. Because if she didn’t have the pipe, she’d just be a normal person and she is a normal person now. It’s just that she has… medical reasons’	Sibling of a child with a congenital condition
**Spiritual and existential concerns**		
Q10	‘I think its variable. It’s um, I think sometimes it’s not necessarily a question that we are very good at asking. I think it’s one that we miss out on.’	Nurse
Q11	‘…as I’ve gone through all of these…all of this and I’ve been in hospital…erm I always remember that, you know there as someone who suffered even worse for me and that, you know gives me peace because I know that you know I can suffer…you know I can go through all these things but nothing is gonna like keep me down and that yeah I’m always gonna continue to get back up on my feet and even if…even if something happens that, you know I’m in hospital for a very long time and things don’t get better, I know that you know, that there’s a greater hope and like the greater hope is in Jesus and that I trust in that. You know even whatever happens, whether you know I die or whether I live, it’s for “him” and you know I’m just gonna continue to live a life according to his grace’	17-year-old, gastrointestinal condition
Q12	‘I’m just thinking about parents that…that talk about usually losing their faith actually when it comes to end of life. I mean some find their faith and some lose it’	Psychologist
Q13	‘I’m not godly, I don’t believe that there’s a higher being out there I don’t believe anything like that but I’m not a hundred percent certain and I just felt it was the right thing to do because I got told that my son was gonna die. I need to get him christened just in case’	Mother of a 14-year-old with metabolic condition
Q14	‘So, in [country], if you’re [tribe] if someone dies, someone stays with the body until they are buried. And that is built into the system. But here if [child] was to die in hospital either after hours or a weekend or bank holiday, the body would be moved to the morgue alone and I wouldn’t be able to be with him until a death certificate was issued, which can only be done by a person who works in the morgue who isn’t want to be there on a bank holiday, after hours or on a bank holiday weekend. Um so we have it in our care plan that [child] is not to die in hospital.’	Mother of a 2-year-old with a metabolic condition
Q15	‘They haven’t told me, after the year, they don’t know if I’m going to live or everyone knows what’s the other, they’ve said they can only tell what is going to happen now.’	13-year-old, cancer
Q16	‘The teenager that died recently, I mean she was still going to do her GCSE’s this summer. And she died much quicker than we thought. But no, she was definitely going to still do them.’	Nurse
Q17	‘…just …remember that even if I have this disease, I want to live my life normally and it will get better. I mean the treatments already started so now I will get more en…I will have more energy and I’m looking forward to just enjoying what…what is coming’	15-year-old with metabolic condition
Q18	‘So, I dreamt of you know doing having the lifestyle with [child] like I’d had. Being a beach bum, you know sort of rock pooling. And you know sort of that, and you know you had all these dreams and aspirations and things. But they didn’t pan out’	Mother of 10-year-old with neurological condition
Q19	‘You know ‘why me?’ and we had a lot of anger first off, again the issue I just said ‘Oh you know ‘eat your veg, fruit and veg, you know you’ll be big and strong’ you know’, ‘drink lots of water because it’s good for you’ erm…and initially we had the “well you lied to me, why…you know why, why me. Why, what have I done wrong?”’	Father of 13-year-old child with a gastrointestinal condition
Q20	‘It’s I guess it’s not about you know, her, her being, you know, her physical, you know if she’s if she has physical issues. It’s more I guess about her learning and development you know. Making sure that she can, not necessarily develop at the same pace as everyone else but she’s still developing. So that you know, hopefully she can you know, she can experience love, relationships, work and you know, she has you know what we consider to be the standard things.’	Father of a 1-year-old child with an infectious disease

**Table 4:** Participant quotes—emotional and psychological concerns, and social concerns

**Emotional and psychological concerns**		
Q21	‘I can’t do as much as other people. I can’t go out as often. You know… I can’t um… go and hang out with friends or go to the town because… I get worn out quickly. And if something was to happen to me no one would know what to do.’	14-year-old with a congenital condition
Q22	‘I mean she works in (child’s) old school on a Saturday now, she’s got a Saturday job down in err (area in London) and they said to her ‘you know, what you know…is there things that you wouldn’t want to do for the…’ she said ‘I’ll do anything’ she said ‘you know I’ll…I’ll change their pads’ she said “I…I will do anything that makes them happy, to get a smile out of them or to just know that I am helping them”’	Mother of a 3-year-old child with a neurological condition
Q23	‘We haven’t met that many with the same sort of symptoms… and I think it’s good for (child) to see that… its good for us to meet other families I think’	Mother of a 15-year-old child with a neurological condition
Q24	‘It’s a long, sometimes painful, sometimes heart-breaking but it’s an ocean of emotions that you go through. You’re in this boat and it’s your diagnosis with you and imagine you’re in this boat, you’re in this ocean of emotions and that boat is your diagnosis, the boat sometimes breaks apart but you’ve just, you just have help from the sunlight’	13-year-old with cancer
Q25	‘It’s hard work, its hard you know for the whole family. It has an effect on everybody, because everyone’s trying to help and everyone’s worried and you know trying to also make sure she’s okay and so it is…it does, it is…it affects everybody in the family definitely.’	Mother of a 4-year-old with a congenital condition
Q26	‘I: What would you say are your main care and support needs for (child)? P: For (child)… is that he’s happy and safe and that he has an enriched life as much as possible’	Mother of a 12-year-old with a congenital condition
Q27	‘We had a young girl who, she couldn’t go to the bathroom on her own…umm at the end and she wanted the carers to take her rather than her mum and it was because she was a 14-year-old girl and she just wanted that…and her mum was very, she was a little bit upset by it initially…erm because her mum just wanted to do everything for her’	Commissioner
Q28	‘I can’t really have that much privacy because we don’t know whether or not I’m going to have a seizure or not’	17-year-old with cancer
Q29	‘….it is a bit strange just sort of often having so many people in your house. Erm, it does feel a bit of a loss of sort of privacy but, again, that’s just something that we’ve got used to really.’	Mother of an 8-year-old with a congenital condition
Q30	‘I don’t always talk to my Mum, I don’t like talking to her because I don’t like making people upset or anything like that of how I am feeling.’	15-year-old with cancer
Q31	‘And my husband did see, my husband saw [psychologist] here for a little while. But again, he found it really tricky, because he’s not, he only comes in on a weekend cause he started seeing her when he was off, when she was initially ill. But he went back to work so he couldn’t get up to see her.’	Mother of a 12-year-old with cancer
Q32	‘One of the young people who we lost quite recently, the carers just supported mum to do things like make a memory box and just sit and read stories with the young person and it was just giving the young person and the family those memories really.’	Commissioner
**Social concerns**		
Q33	‘It’s definitely affected my social life because I spent most of the year in hospital receiving my chemotherapy and radiotherapy so I wasn’t able to go to school’	17-year-old with cancer
Q34	‘Okay…erm so the education and the provision of education in its broader sense for children with special needs and how the cur…you know it doesn’t feel like the current system is set up for children to achieve their potential. ….So, we spent an enormous amount of time ensuring that he gets the right provision in terms of education and associated therapy services, you know so physio, OT, speech and language all that sort of stuff…erm but that’s a constant battle and dealing with the local authority is absolutely exhausting because they can’t…don’t function.’	Father of an 8-year-old with a congenital condition
Q35	‘Erm so personally I found socially, I really, really felt isolated…erm for quite a long time…erm tried to find places to take him, groups to go to…’	Mother of a 3-year-old with a neurological condition
Q36	‘I just miss like [pause] the environment of school and like, talking with people, because it gets lonely as well’	15-year-old with a gastrointestinal condition
Q37	‘I: So, how do you manage those expectations? P: I think it’s being honest. I think it’s telling them what can be expected…umm that there are times when you might be a bit behind getting all these things and the reason why you will be, is about being safe but that you will get there.’	Nurse
Q38	‘Um and it’s difficult to trust people because, erm particularly er considering that we’ve had quite an adversarial relationship with our local authority at times, um then you’re not always completely sort of clear erm how independent people are and who’s on your side.’	Mother of an 8-year-old with a congenital condition
Q39	‘It’s very much a full time job for me. And I’ve, I had to give up my job… and I’ve never worked as hard as I am now.’	Mother of a 4-year-old with a metabolic condition
Q40	‘Yeah, and some…and you wouldn’t believe how many people I see funding stuff themselves. ‘How much is this, how much is that? Do you know, if its broken, how do we get it repaired…umm it needs a service, do you…can I have the number for the service of you know the suction machines’. And I think, goodness why are you paying for this stuff yourselves?’	Mother of a 4-year-old with a congenital condition

**Table 5:** Participant quotes—practical concerns and normality

**Practical concerns**		
Q41	‘I was absolutely terrified that she’d go to hospital and either, one die in hospital which we don’t want or two they do things to her that we didn’t want to happen. So, I never took her to hospital, just kept her out and then when they…once they did the DNR and…and all of our wishes…erm that’s when I…I felt more comfortable to be able to take her in.’	Mother of an 8-year-old with a neurological condition
Q42	‘I had a parent who said to me, ‘(participant) you said we have a choice, we don’t have a choice. The choice…the choice isn’t there’ and that’s because a hospice refused to take a patient with a central-line and the parents did not want the sub cut line’	Nurse
Q43	‘Sometimes some very…you know people just don’t die overnight, children just don’t die overnight or often don’t die within a couple of days. They have a…you know a trajectory that’s days to weeks, to months sometimes and actually, for the parents to be able to deliver, we expect parents to do a lot these days and we have more and more gaps and you know we sometimes need to plan around the fact that we don’t have anybody who could go out to change a pump.’	Doctor
Q44	‘I think the family stuff, they do get more concerns as they get older. When they are older and bigger its more stress and pressure… physically on the parents and carers.’	Nurse
Q45	‘Umm…yeah its…its fairly frequent, yeah (wife) tends to book the…book the respite hours…err yeah and we…I mean (child’s) we’ll have the respite care and we’ll have a long weekend, well not a long weekend…err maybe from Friday through to Sunday and that enables us to go and take (sibling) out and sort of do normal…yeah normal sort of family things… it’s not often we do stuff as a four, you know a foursome, because he is so difficult to manage or take him out…’	Father of a 12-year-old with a congenital condition
Q46	‘I: Out of everything what do you think matters most to you? P: Getting home.’	12-year-old with cancer
Q47	‘I: And how do you feel when you’re in hospital? P: Well, I’m happy because I get better, but then I’m sad because I miss school, miss my friends, miss my family, yeah’	12-year-old with a respiratory condition
Q48	‘I: So, do you have any questions about your illness and how you are cared for? P: Uh, I know pretty much what happens and things like that and what will happen. So not really…’	15-year-old with cancer
Q49	‘I: Is there anything else you want to tell me about when [brother] was in hospital? And what you thought, what they told you? P: Mmmm, no thank you. They didn’t really tell me anything. I: What, no one told you what’s going on? P: They didn’t tell me what was going on, but they did tell my parents. I: Yeah. Do you wish they did tell you what was going on? P: Yup.’	Sibling of a child with a metabolic condition
**Normality**		
Q50	‘P: No, it was um just about not caring about my condition. Just ignoring it. I: So, just ignoring your condition and do you think that’s just because you want to forget about it? P: No, I don’t really, I don’t really care about it. I don’t really let it get in my way so….’	11-year-old with neurological condition
Q51	‘I was just brought up like this. I don’t really remember anything different.’	14-year-old with a congenital condition
Q52	‘So, I started going back to school a little bit and my mum…I just want…because I just love school. I just wanted to go back and get back to normal and everything and then my mum was like, ‘okay just like do half days’ and everything and I was like, ‘no please let me do a whole day’. I was like (laughter) begging her to do it’	13-year-old with a gastrointestinal condition
Q53	‘I want to be a normal person. Sure, normal is a harsh word that some people may not like using, oh my gosh I can’t believe this person is using this word, but what other words could I use’	13-year-old with cancer
Q54	‘I often, yeah, I do feel worried about things. I think mostly, I’m more worried about my normal… like going back to normal. I really want to just be normal, I’m just scared that the more time I spend in hospital, the less I’m normal, the less I’m gonna be like all the other kids my age, yeah’	17-year-old with cancer
Q55	‘Erm and but back then about 18 months ago I asked her [doctor], I said, you know ‘is he really poorly?’. You know I couldn’t grasp it because giving him these recovery meds, it was just run of the mill, it’s what we did you know. And I am thinking is he really poorly? And she [doctor] said- ‘The only reason that [child] is still here is because of the amount of medication he’s on’. But erm you know and her making me realise that this is not the norm you know. There aren’t kids in the community having this level, kids that need this level of medication are generally in hospital.’	Mother of 10-year-old with neurological condition
Q56	‘…sometimes you see like, when you…when like you’re at the park or something, like you see people staring and you just think…oh honestly, I couldn’t really care any less. Because if she didn’t have the pipe, she’d just be a normal person and she is a normal person now. It’s just that she has… medical reasons’	Sibling of a child with a congenital condition

### Physical symptoms and concerns

All participants spoke of the importance of managing pain and other physical symptoms (such as seizures and infection), and the impact of multiple medical interventions. Symptom management and children being ‘comfortable’ was important to parents and professionals (T3Q1). Pain and other symptoms were often linked to other themes. For example, if physical symptoms were well managed, then children were more likely to be happy, have reduced anxiety, and be able to participate in normal childhood activities. Professionals discussed symptom management in relation to managing expectations of care and setting realistic goals (T3Q2). Seizures were particularly distressing and often described as difficult to manage by parents (T3Q3), sometimes being triggered by noise and over excitement (T3Q4), meaning siblings had to play quietly.

Participants from all groups spoke of the difficulties children had with eating and drinking. Some children described feeling under pressure to maintain weight (T3Q5), and others required artificial feeding. Healthy siblings spoke of feeling guilty about consuming treats in front of a sibling who was unable to eat (T3Q6).

Tiredness and fatigue were a concern for both children and parents. Parents spoke of lack of sleep and exhaustion which impacted on ability to care for their child (T3Q7). Children spoke of overwhelming fatigue causing lack of stamina and the need to take daytime naps (T3Q8).

Siblings and children with life-limiting conditions were very aware of changes in physical appearance which impacted on school attendance, seeing friends, and social activities (T3Q9).

### Spiritual and existential

Professionals spoke of lack of confidence in discussing spiritual and existential issues (T3Q10). For some patients and families, faith offered a source of comfort (T3Q11, S1Q1), whereas for others, it was a potential cause of conflict (T3Q12). Some moved more towards faith, for example, by having their child christened ‘just in case’ (T3Q12). Faith was also important in decisions about future care, with one participant describing how hospital policy on death registration and care of the body conflicted with her own culture (T3Q14).

Participants from all groups spoke about the uncertainty surrounding length of life (T3Q15), with children wanting to plan for their future regardless of their prognosis (T3Q16). Children were often determined to overcome and survive (T3Q17, S1Q3). Parents spoke of adjusting their hopes and dreams for a child who would be unlikely to reach typical life-course milestones (T3Q18) and questioned the meaning of illness (‘why me/why my child?’) (T3Q19). They expressed a desire for their child to live life as fully as possible, to their full potential, experience relationships with others, and have things to hope for and look forward to (T3Q20).

### Emotional and psychological

All participants described many psychological and emotional impacts of living with a life-limiting condition. Where children had been diagnosed during childhood, rather than at birth, they spoke of an awareness of being different and having different life experiences (T4Q21). For some siblings, their experience led to desires to pursue caring careers (T4Q22), while children with life-limiting conditions sought out others with similar experiences (T4Q23).

All participants spoke of the life-altering impact of living with a life-limiting condition (T4Q24). They described anger, worry, sadness (T4Q25, S1Q4), and an overwhelming desire for children to be happy (T4Q26, S1Q5). Older children spoke of loss of privacy, control, and independence (T4Q27–28, S1Q6–7). Parents also faced a loss of privacy due to having professionals in their home, and the wish to maintain some control over their child’s care and condition (T4Q29, S1Q8–9).

There was a sense of children and parents wanting to protect each other from how they were feeling, specifically around discussion of prognosis (T4Q30). Parents found accessing psychological support for themselves and siblings challenging, as this is often hospital-based and does not fit around work and school hours (T4Q31). Individuals also spoke of the importance of memory making (T4Q432).

### Social concerns

Children were focused on being able to undertake usual childhood activities such as seeing friends, pursuing hobbies, and playing. School was important to parents and children for maintaining friendships, retaining a sense of normality and planning for a future by preparing for exams (T4Q33, S1Q10). Parents spoke of difficulty in accessing suitable education for their child due to complex medical needs (T4Q34). Many parents and children experienced loneliness and isolation due to absence from school and not being able to find suitable activities for their child to take part in (T4Q35–36, S1Q11). Unclear communication about symptom management goals and service availability often led to unrealistic expectations, causing discord between professionals and families. This impacted on decision-making, trust and respect, and continuity and co-ordination of care (T4Q37–38, S1Q12).

Parents and professionals spoke of the financial impact of having a child with a life-limiting condition in terms of having to give up work, the expense of hospital stays, and self-funding equipment due to lack of availability (T4Q39–40, S1Q13).

### Practical concerns

Parents and professionals were concerned with many practical aspects of care. These included care quality, advance care planning, service availability and facilities (T5Q41–42, S1Q14), the huge familial burden of care, and the logistics of managing this (T5Q43, S1Q15). The physical burden of care increased as children grew older (T5Q44). Access to respite care was essential to many parents of children without a cancer diagnosis, allowing them to have uninterrupted sleep and spend time with other children (T5Q45). Children did not share these concerns and were more interested in being at home (not hospital), being able to see their friends and carry on with their usual activities (T5Q46–47, S1Q16–17).

Parents and children felt well informed about the condition, treatment, and available services, which was considered important (T5Q48, S1Q18). Siblings often felt less well informed and not included in care (T5Q49).

### Normality

The theme of normality was cross-cutting across all other themes. Children wanted to live life as normally as possible, focusing on being a child first, with their condition secondary to this (T5Q50). They described the importance of seeing friends, attending school, and making plans for the future. To achieve this, physical symptoms need to be well managed. Children with varying diagnoses described normality in different ways, with all wanting to pursue normal childhood activities. When a condition had been present since birth or soon after, children spoke of feeling normal and not knowing any different (T5Q51). Those that had been diagnosed later in childhood spoke of having to adjust to a new normal such as having carers in the home (T5Q52). Those with an uncertain prognosis, such as cancer, wanted life to return to pre-diagnosis normality and desired to be like their healthy peers (T5Q53–54). Parents who had been caring for a child with a life-limiting condition for many years had often adjusted to their child’s care needs and had to remind themselves of their unique situation (T5Q55, S1Q19). Siblings spoke of seeing their unwell sibling as normal but with different needs (T5Q56, S1Q20).

## Discussion

This study provides novel evidence of inter-related symptoms, concerns, and care priorities for children with a wide range of life-limiting conditions and their families, from the perspectives of multiple stakeholders (including children). This is an area of knowledge not previously well described [21]. Symptoms and concerns were broadly the same across the spectrum of life-limiting conditions, which is a finding previously reported [21, 32]. Most were evident across participant groups, except practical aspects of care, which were not a priority for children.

The concept of child-centred care encourages healthcare professionals to place the child and their interests at the centre of thinking and, where able, include them as active participants [22]. The focus of care is on the child in the context of the family, while acknowledging the child’s wider environment and relationships [22, 33]. Previous studies have found that children with cancer and their families try to adjust to a ‘new normal’, and those with severe neurological impairment were able to regain some normality with input from a paediatric palliative care team [34–36]. Our study adds to the concept of pursuing normality within the context of children living with life-limiting conditions, demonstrating that a child-centred approach to care needs to take an individual and holistic view of the child, ensuring that physical, emotional, social, practical, and spiritual concerns are addressed. This enables children to pursue normal childhood activities such as attending school and seeing friends. Children in our study wanted to be seen as children first, with their condition coming second to this, reinforcing that children do not want to be defined by their condition [37].

We found children wanted the opportunity to make plans for a longer-term future, even if these would not be realised, adding to the concept of pursuing normality. In contrast, a previous study found that children with neuro-disability only want to plan for the present or near future [38]. This difference may be due to the older age of the sample of participants with neurodisability meaning they had a better understanding of their condition. The heterogeneity of conditions in our study may also have contributed to our finding, as curative treatment for some life-limiting conditions is feasible, but may fail [39].

Taking a child-centred approach to care for children with life-limiting conditions needs to incorporate support for the family, while ensuring that the child remains the focus of care [40, 41]. This is important for families of children with life-limiting conditions, as this study demonstrates that they often have to provide complex, burdensome care. Many life-limited children are unable to communicate their needs due to their condition, and parents will need to advocate for their best interests. Parents require access to adequate holistic services, particularly respite care and practical support to enable them to provide care. Parents and siblings need time and space to undertake their own normal activities such as self-care, spending time as a family, and seeing friends. In our study, this was not always achieved, with insufficient or inaccessible practical, psychological, educational, and respite support often highlighted, along with lack of co-ordination and communication between services. To attempt to address this pursuit of normality and accomplish child-centred care, services need to be co-ordinated around child and family needs [40, 42], and this should be considered in the design of future health services for those with life-limiting conditions.

In our study, we found that children as young as five wanted to be informed about their condition, supporting a child-centred approach to care where the child is, where able, encouraged, and supported to be an active participant. Other studies have found that the desire to be informed about a condition is associated with adolescence, rather than younger children [21]. Siblings wanted to be informed, which is a finding previously reported in children whose parents have a life-limiting illness [43].

### Strengths and limitations

As far as the authors are aware, this is one of the largest studies conducted exploring symptoms and concerns of children with a range of life-limiting conditions from multiple stakeholder perspectives. We have demonstrated that verbal children from the age of five years old are willing and able to participate in research and share their perspectives on their condition. This study’s strengths include our large sample, wide range of stakeholder participants, and the range of life-limiting conditions. Fathers, who are often underrepresented in palliative care research, represented 25% of our parent sample [44].

Our study has several limitations. Recruitment took place in a small number of UK sites and data on ethnicity was not collected. One site recruited only children with gastrointestinal diagnoses, and this is reflected in the higher number of participants from this group. There are almost 400 different life-limiting conditions known to affect children, so not all could be included [12]. Many children with life-limiting conditions are non-verbal and cannot meaningfully share their perspectives and parent/proxy-reporting has to be used. The findings presented here reflect those of children who were able to participate. As a child-centred approach to care should include support for the family, care must enable them to use their knowledge and experience of their child in order to advocate for them. The child’s needs and interests should always be at the centre of care and decisions [42].

### Clinical and research implications

This study provides a comprehensive insight into what symptoms, concerns, and care priorities are important to children with life-limiting conditions and their families, to enable healthcare professionals to support them to be viewed as children, rather than their condition, within a child-centred model of care. We have demonstrated that children can be meaningfully involved in such studies [45]. Findings will be used to develop the construct for a valid child-centred outcome measure for use in this population.

## Conclusions

Children want to focus on pursuing normal childhood activities, but need a holistic approach in addressing their care needs to achieve this. Improvements in accessibility, availability, and co-ordination of relevant health services are required.

## Supplementary Information

Below is the link to the electronic supplementary material.

Supplementary file1 (DOCX 26 KB)

## Abbreviations

Children: Children and young people
Healthcare professional: Health and social care professional
Life-limiting condition: Life-limiting and life-threatening condition
Parents: Parents and carers

## Declarations

### Disclaimer

Principal Investigator: Richard Harding. This article reflects only the authors’ views and the European Research Council is not liable for any use that may be made of the information contained therein. Fliss Murtagh is a National Institute for Health Research (NIHR) Senior Investigator. The views expressed in this article are those of the author and not necessarily those of the NIHR, or the Department of Health and Social Care.
