# Supplementary material for: Achieving child-centred care for children and young people with life-limiting and life-threatening conditions—a qualitative interview study
Source: Eur J Pediatr. 2022 Aug 12;181(10):3739–52. doi: 10.1007/s00431-022-04566-w (PMC9371630; doi:10.1007/s00431-022-04566-w)
Supplement: Supplementary file 1 — Supplementary file1 (DOCX 26 KB) [file 431_2022_4566_MOESM1_ESM.docx]

Supplementary table 1

| **Quote**  **Number** | **Quote** | **Participant details** |
| --- | --- | --- |
| **Spiritual and existential concerns** | | |
| Q1 | “We do occasionally go up to the chapel and she’s been up and stuff. And I’ll often go up and spend some time on my own.” | Mother of a 12-year-old with cancer |
| Q2 | “P: Well, I do even though I’m gonna be a Police  I: You’re gonna be the Police, are you? Why do you want to be in the Police?  P: Umm…because I want to…erm like…umm have a big job arresting people” | 5-year-old with gastrointestinal condition |
| Q3 | “With cancer you want to live, you want to show people that you can overcome this” | 13-year-old with cancer |
| **Emotional and psychological concerns** | | |
| Q4 | “It makes me feel upset because erm…when I talk about him, it’s kind of like I feel… I feel like I’m the unusual one at school” | Sibling of a child with a gastrointestinal condition |
| Q5 | “I: What would you say are your main care and support needs for (child)?  P: For (child)… is that he’s happy and safe and that he has an enriched life as much as possible” | Mother of a 12-year-old with a congenital condition |
| Q6 | “And a couple of them have actually asked the parents to call an ambulance right at the end and died in hospital. I think it was because they were scared, and they didn’t want their parents to have to struggle on.” | Nurse |
| Q7 | I: Do the nurses ever come and wake you up in the night?  P: Yeah!  I: Yeah, do you like that?  P: No! | 6-year-old with a gastrointestinal condition |
| Q8 | “I think for us it’s important that we live very sort of separate lives to a degree… one of us will do something with (sibling), someone does something with (child) and vice versa, so (partner) and I are never a couple, and I think yeah, that needs to be changed” | Mother of a 12-year-old with a congenital condition |
| Q9 | “I think this year has been a big thing with her not being right and not knowing and not being able to do anything to help has been horrible, because it’s been totally taken out of our hands and out of our control. So, yeah that’s probably one of our biggest concerns, the not knowing and not being able to be in control of it both” | Mother of a 13-year-old with a metabolic condition |
| **Social concerns** | | |
| Q10 | “I was crying at some of the exams because like, I’m very like, a very studious and like [pause] I like to achieve like high grades and that so, just [pause] disappointing” | 15-year old with gastrointestinal condition |
| Q11 | “P: Sometimes in the night…some…sometimes I have the separate bedroom from (sibling) and (mum) is…is three bedrooms away from mine, so I feel very alone  I: Ah, so you like to be in the bedroom with (sibling)?  P: Yeah” | Sibling of 8 year old with a congenital condition  (talking about staying in a hospice) |
| Q12 | “There is also the families that are quite, understandably they are going through a really stressful situation, but they can be sometimes quite obstructive and difficult as well. So I think that that sometimes impacts communication because if you’re, for example, very obstructive, we have an obstructive grandmother at the moment who is there all the time and actually the nurses are really nervous about talking to her because they’re not too sure what way she’ll go either time, so as a result of that, that impedes the communication and also impacts patient care to some extent as well. So, I think that’s a real challenge.” | Nurse |
| Q13 | “Yes and there’s all the erm…hospital appointments as well, so you’re driving there all the time. The one in London, and I mean if you’re paying for that hotel every time as well and it’s kind of…yeah a trip to London’s probably and an overnight with everything surrounding and if you count driving there, because I drive to the hotel. It’s probably about five hundred pounds a time. So, you have to think actually, you know you….” | Mother of a 15-year-old with a neurological condition |
| **Practical concerns** | | |
| Q14 | “…yes when she…she had erm…a five-night telemetry…erm last time at the inpatient there and but they had just…there was a…erm kind of a bench on the side of her room so we could sleep on there. It was hot, it was hot, the air conditioning wasn’t working.” | Mother of a 15-year-old with a neurological condition |
| Q15 | “We are basically sort of managing the care ourselves from home but there’s a lot of emails and phone calls going around with all the doctors.” | Mother of a 4-year-old with a metabolic condition |
| Q16 | “I: And how do you feel when you’re in hospital?  P: Well, I’m happy because I get better, but then I’m sad because I miss school, miss my friends, miss my family, yeah” | 12-year-old with a respiratory condition |
| Q17 | “I: And what sort of things do you like doing?  P: I like erm…playing football and I like playing lots of games and I like to read a lot” | 5-year-old with a gastrointestinal condition |
| Q18 | “When I do have questions I always find someone who can answer it. Or if the people I’m asking can’t answer it, again I’ve got a really good team who will go away and say I will get back to you and they do. You know with anything I have to ask so, I don’t, I wouldn’t say I have a problem with that.” | Mother of a 14-year-old with a metabolic condition |
|  |  |  |
| **Normality** | | |
| Q19 | “because you have no real feel for erm…where your child sits with these things, you know like a lot of…a lot of them…a lot of other children that have night packages, they have it because their children are on oxygen or…or whatever they’re on at…at night and like (child) is…is relatively mild…mild…he’s very complex, but I think, oh well they’re just doing medicines all night and milk and then you think, actually yeah that’s every two hours. It’s a medical mission and…and then he has his breath holds as well which are quite dangerous and you kind of think, oh yeah okay I…I can see why we get it now” | Mother of a 3-year-old with a neurological condition |
| Q20 | “…sometimes you see like, when you…when like you’re at the park or something, like you see people staring and you just think…oh honestly, I couldn’t really care any less. Because if she didn’t have the pipe, she’d just be a normal person and she is a normal person now. It’s just that she has… medical reasons” | Sbling of a child with a congenital condition. |
